# Comparative assessments of myocardial iron load in thalassemia patients between dark blood and bright blood MRI T2* techniques

**DOI:** 10.3389/fmed.2025.1608870

**Published:** 2025-07-31

**Authors:** Jixing Yi, Min Wu, Suzhen Wei, Qiliang Huang, Bumin Liang, Peng Peng, Tao Li, Fengming Xu

**Affiliations:** ^1^Department of Radiology, Liuzhou Worker’s Hospital, Liuzhou, China; ^2^Department of Gastroenterology, Liuzhou Worker’s Hospital, Liuzhou, China; ^3^School of International Education, Guangxi Medical University, Nanning, China; ^4^Department of Radiology, The First Affiliated Hospital of Guangxi Medical University, Nanning, China

**Keywords:** cardiac magnetic resonance, dark blood, bright blood, myocardial T2*, thalassemia

## Abstract

**Objective:**

To investigate the difference of black blood (DB) and bright blood (BB) T2* techniques at 1.5 T and 3 T in the assessment of myocardial iron load in patients with thalassemia (TM).

**Methods:**

As a retrospective study. CMRtools software was used to measure myocardial T2* in 359 patients with moderate (60 g/L < Hemoglobin<90 g/L) or severe (Hemoglobin<60 g/L) thalassemia. A truncation method was used to remove signal values that deviated from the fitted curve. T2* (DBx-T2*, BBx-T2*) containing all (eight echoes) signals (DB8-T2*, BB8-T2*) and the optimal signal (coefficient of determination *R*^2^ > 0.95) were recorded. The difference, correlation and consistency of T2* measured by different methods were compared.

**Results:**

There was no significant difference (*p* > 0.05) in myocardial T2* measured by different methods (1.5 T, 3 T), and all of them were highly positively correlated (*p* < 0.05, r_s_ > 0.9). Bland–Altman analysis showed that (1.5 T) DB8-T2* and DBx-T2*, DBx-T2* and BBx-T2* had good consistency (*p* > 0.05). (3 T) DB8-T2* and DBx-T2* had good consistency (*p* > 0.05). There were proportional differences in T2* values measured by the other methods (*p* < 0.05), and none of them could be considered to have good consistency.

**Conclusion:**

DB CMR T2* and BB CMR T2* can be interchangeable in the assessment of myocardial iron load in TM patients. However, DB CMR T2* is more stable and reliable.

## 1 Introduction

Thalassemia (TM) is a anemia caused by globin chain synthesis disorder caused by mutations in the globin gene (1). Due to ineffective erythropoiesis leading to varying degrees of anemia, the vast majority of severe TM patients inevitably require long-term transfusion therapy (2). Long-term transfusion therapy will lead to myocardial iron overload in patients, which will cause a series of adverse cardiovascular events, such as malignant arrhythmia, acute cardiac function damage, myocardial fibrosis, etc., and eventually progress to heart failure, which is one of the main causes of death in patients with severe TM (3). Patients with myocardial iron overload need timely iron chelation therapy, whether it is the assessment of iron load before iron chelation or the efficacy evaluation after iron chelation. There is a need for an early, accurate and stable method to assess myocardial iron load and prompt intervention. Chemical measurement of iron concentration after endocardial biopsy can accurately determine the patch concentration at the biopsy site, but it is a traumatic examination, the iron deposition in the heart is not uniform, and the endocardial iron content cannot fully represent the myocardial iron content, so it is difficult to widely apply endocardial biopsy in clinical practice (4, 5).

T2* technique based on MRI gradient echo (GRE) imaging sequences has been established as a non-invasive standard method for quantifying organ iron load (6–10). By measuring T2* of the corresponding organ, clinical non-invasive assessment of organ iron load can be achieved. Cardiac magnetic resonance (CMR)T2* technology, as a non-invasive and convenient method to quantify myocardial iron content, has high sensitivity, and plays an important role in assessing the myocardial iron load and the risk of cardiac complications, and also in the regular follow-up. It has become an important nursing standard for patients with myocardial iron overload disease worldwide (11–13). Bright blood (BB) and dark blood (DB) techniques are commonly used in CMR examination (14, 15). BB CMR can be divided into flow-dependent and non-flow-dependent techniques according to the different flow-dependent characteristics, which is beneficial to the display of lumen morphology. DB CMR can display high signal on the arterial wall by inhibiting the blood pool signal, which can highlight the myocardial structure, morphology and signal, and then carry out clearer observation and analysis (16).

Is there any difference between DB CMR and BB CMR in the assessment of myocardial T2* measurement (myocardial iron load), and which technique has higher stability and reliability in the assessment of myocardial iron load? The aim of this study is to analyze the images at different magnetic fields (1.5/3 T) with DB and BB CMR T2* in TM patients, and to compare the quantitative assessment of myocardial iron deposition between DB and BB CMR T2* in TM patients.

## 2 Materials and methods

### 2.1 General information

The data of 869 patients with moderate (60 g/L < Hemoglobin<90 g/L) or severe (Hemoglobin<60 g/L) thalassemia who underwent cardiac iron quantification on MRI in Liuzhou Workers Hospital, the First Affiliated Hospital of Guangxi Medical University, and Guangxi Medical University Tumor Hospital from January 2011 to December 2022 were consecutively selected and retrospectively analyzed. Inclusion criteria: (1) thalassemia was confirmed by genetic diagnosis; (2) DB and BB T2* MRI were performed to quantify cardiac iron. Exclusion criteria: (1) Large image artifacts; (2) complicated with other heart diseases. A total of 359 patients (we have de-identified all patient details) were enrolled in the study, including 245 males and 114 females. The age of patients ranged from 4 to 63 years, with a median age of 18 years. This study was conducted in accordance with the Helsinki Declaration of 1975 as revised in 2024 and approved by the Ethics Committee of Liuzhou Worker’s Hospital, Liuzhou (No. LW2023039. Nov. 07, 2023) and the Ethics Committee of the First Affiliated Hospital of Guangxi Medical University, Nanning (No. 2023-E548-01. Oct. 09, 2023). The reporting of this study conforms to STROBE guidelines (17).

### 2.2 MR image acquisition

359 patients underwent Siemens 1.5 T MRI scanner (Magnetom Avanto Fit and Altea, Siemens Healthcare, Erlangen, Germany) and Philips 1.5 T MRI scanner (Achiva, Philips Medical Systems, Best, Netherlands). Before the MRI examination, the patient was repeatedly trained in inhalation, exhalation, and apnea; the patient was asked to calm down and hold his/her breath at the end of the expiration during the scan. Firstly, the chest cross-sectional localization scanning was performed with Turbo FLASH sequence to locate the cross-sectional image; the left ventricular long-axis image parallel to the ventricular septum was obtained, after which the left ventricular short-axis image perpendicular to the ventricular septum was obtained. Then, the long-and short-axis images of the left ventricle parallel and perpendicular to the ventricular septum were located, and a single-breath-hold cine sequence was scanned to obtain continuous left ventricular short-axis cine images from the left atrial ventricular junction to the apex of the heart. The left ventricular outflow tract cine images were obtained by locating the aortic plane perpendicular to the short axis of the left ventricle and the long axis of the left ventricle parallel to the ventricular septum. The papillary muscle in the middle of the left ventricular septum was scanned by multi-echo gradient-echo (GRE) sequence to obtain images based on which the cardiac T2* values were measured quantitatively. BB T2* sequence scanning parameters: flip angle (FA): 20°, matrix (MA): 224 × 112, repetition time (TR): 138.00 ms, echo time (TE): 2.97–21.68 ms, respectively 2.97, 5.54, 8.23, 10.92, 13.61, 16.30, 18.99, 21.68 ms, field of view (FOV): The slice thickness was 10.0 mm, and the slice spacing was 2.0 mm. DB T2* sequence scanning parameters: FA: 20°, MA: 224 × 112, TR: 268.00 ms, TE: 1.84 to 20.55 ms, respectively 1.84 ms, 4.41 ms, 7.10 ms, 9.79 ms, 12.48 ms, 15.17 ms, 17.86 ms, 20.55 ms, FOV: The slice thickness was 10.0 mm, and the slice spacing was 2.0 mm.

106 patients were simultaneously scanned with a Siemens 3 T MRI scanner (Verio, Siemens Healthcare, Erlangen, Germany). Parameters: BB T2* sequence scanning parameters: FA: 20°, MA: 256 × 128, TR:138.00 ms, TE: 2.97–21.68 ms, respectively 2.97, 5.54, 8.23, 10.92, 13.61, 16.30, 18.99, 21.68 ms, FOV: The slice thickness was 10.0 mm, and the slice spacing was 2.0 mm. DB T2* sequence scanning parameters: FA: 20°, MA: 256 × 128, TR: 268.00 ms, TE: 1.84 to 20.55 ms, respectively 1.84 ms, 4.41 ms, 7.10 ms, 9.79 ms, 12.48 ms, 15.17 ms, 17.86 ms, 20.55 ms, FOV: The slice thickness was 10.0 mm, and the slice spacing was 2.0 mm.

### 2.3 Image analysis

**Figure 1:**
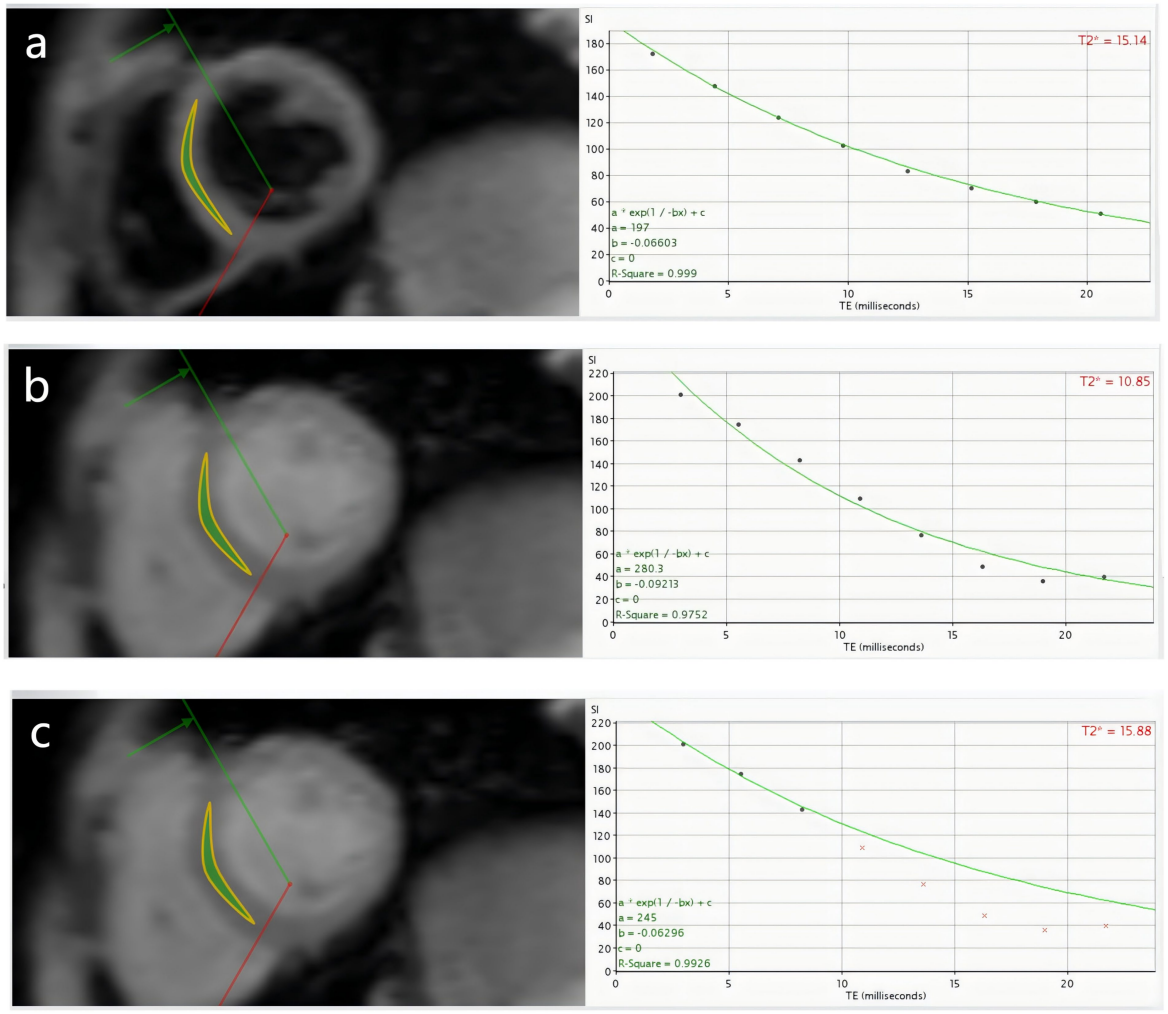
β-TM patient, male, 11 years old, **(a–c)** are DB8-T2*, BB8-T2* and BBx-T2* measured by DB CMR T2* technique, respectively. **(a)** Shows that the 8 echoes of DB sequence were well fitted to a curve, DB8-T2* = 15.14 ms, *R*^2^ = 0.999; **(b)** Shows that the 8 echoes of BB sequence could not fit the curve well, BB8-T2* = 10.85 ms, *R*^2^ = 0.9752; **(c)** Shows the T2*, BBx-T2* = 15.88 ms, *R*^2^ = 0.9926 fitted by the three echoes of the BB sequence after the “truncation” method. To some extent, DB CMR is more stable than BB CMR in quantifying myocardial iron load.

The MRI Data of all patients were processed by CMRtools (CMRtools/Thalassemia Tools 2014, Cardiovascular Imaging Solutions, London, United Kingdom) software. Region of interest (ROI) was delineated along the endocardium and epicardium at the mid-septal level in the short axis plane of the heart. The software automatically calculated the T2* when the full signal was preserved, which was denotedas DB8-T2* and BB8-T2*. In order to compare the stability of the measurement results of different sequences, this study used the “truncation method” to remove the noise signal (7, 8, 18), and recorded the T2* (denoted as DBx-T2*, BBx-T2*, “x” represents the number of signals remaining after the truncation) and the corresponding *R*^2^ when the coefficient of determination (*R*^2^) > 0.95 (Figure 1).

DBx-T2* and BBx-T2* of the 20 patients (Selection by random number method) were measured simultaneously by two radiologists (observer A and observer B) with more than 5 years of experience in cardiac MRI diagnosis to evaluate the inter-observer agreement of measurement results. The DBx-T2* and BBx-T2* values of the 20 patients were measured again by observer A after an interval of 1 week to evaluate the consistency of observer’s measurement results. In the case of consistent agreement between observer measurements, the remaining patient data were measured by observer A and observer B.

### 2.4 Statistical analysis

SPSS 26.0 software was used for statistical analysis. Kolmogorov–Smirnov test was used to evaluate whether the measurement data were normal distribution. Normal distribution data were expressed as mean ± standard deviation (x̅ ± s), and paired *t* test was used to compare the differences between groups. Pearson correlation was used to analyze the correlation between groups. Non-normal distribution data and ranked data were described by Median (M), InterQuartile Range (IQR), maximum and minimum value, and Friedman rank sum test was used to compare the differences between groups. Spearman rank correlation was used to analyze the correlation between groups. Count data were expressed as frequency. Intraclass correlation coefficient (ICC) was used to evaluate the consistency of T2* measured twice by the same observer and T2* measured by different observers (two-way random was selected for “model” and absolute consistency was selected for “type”). MedCalc^®^ statistical software was used to plot the Bland–Altman plots of T2* measured by different methods, and the consistency was analyzed. In this study, *p* < 0.05 was considered statistically significant.

## 3 Results

### 3.1 Intra-observer and inter-observer consistency analysis

The DB8-T2* and BB8-T2* measured by observer A and observer B at the same time were highly consistent: ICC_DB_ = 0.999, 95%CI = 0.998 ~ 1, *p* < 0.0001; ICC_BB_ = 0.997, 95%CI = 0.992 ~ 0.999, *p* < 0.0001. DB8-T2* and BB8-T2* measured by observer A were highly consistent: ICC_DB_ = 0.999, 95%CI = 0.998 ~ 1, *p* < 0.0001; ICC_BB_ = 0.999, 95%CI = 0.998 ~ 1, *p* < 0.0001.

### 3.2 Statistical descriptive indicators of measurement results of different methods

The myocardial T2* and *R*^2^ values measured by different methods did not conform to the normal distribution (*p* < 0.05). The corresponding statistical descriptive indicators are shown in Table 1. By observing and analyzing the statistical indexes of 8 echo and truncated echo *R*^2^ values of the same sequence, it was found that 1.5/3 T-BB8-R^2^ had large fluctuations, and its minimum to maximum values were 0.7533 ~ 1 and 0.5763 ~ 0.9999, respectively, while the corresponding 1.5/3 T-DB8-R^2^ showed a relatively stable fitting. Preliminary results show that DB sequence can quantify the myocardial iron load of TM patients more stably without truncation of deviation signal, and it is not prone to more deviation signal.

**Table 1:** Statistical descriptive indicators of myocardial T2* and *R*^2^ values of thalassemia patients in each group.

Group	Case (*N*)	Median (M)	Quartile P25%	Quartile P75%	Minimum to maximum
1.5 T-DB8-T2*(ms)	359	19.12	11.82	29.76	4.61 ~ 48.12
1.5 T-DBx-T2*(ms)	359	19.26	11.61	29.71	4.61 ~ 48.23
1.5 T-BB8-T2*(ms)	359	18.57	11.40	30.07	3.61 ~ 52.38
1.5 T-BBx-T2*(ms)	359	19.49	11.31	29.53	3.44 ~ 47.92
1.5 T-DB8-R^2^	359	0.9984	0.9965	0.9993	0.9611 ~ 0.9999
1.5 T-DBx-R^2^	359	0.9990	0.9972	0.9997	0.9710 ~ 1
1.5 T-BB8-R^2^	359	0.9917	0.9819	0.9970	0.7533 ~ 1
1.5 T-BBx-R^2^	359	0.9986	0.9960	0.9997	0.9628 ~ 1
3 T-DB8-T2*(ms)	106	9.885	6.1725	14.925	3.14 ~ 37.18
3 T-DBx-T2*(ms)	106	10.175	6.03	15.3175	2.90 ~ 37.02
3 T-BB8-T2*(ms)	106	10.195	6.57	14.725	3.20 ~ 37.12
3 T-BBx-T2*(ms)	106	10.01	5.4725	15.3475	3.18 ~ 37.02
3 T-DB8-R^2^	106	0.9974	0.9937	0.9992	0.982 ~ 1
3 T-DBx-R^2^	106	0.9995	0.997775	0.9999	0.977 ~ 1
3 T-BB8-R^2^	106	0.9865	0.95925	0.99565	0.5763 ~ 0.9999
3 T-BBx-R^2^	106	0.99885	0.9967	0.9997	0.9563 ~ 1

### 3.3 Differences and correlation analysis of measurement results of different methods

**Figure 2:**
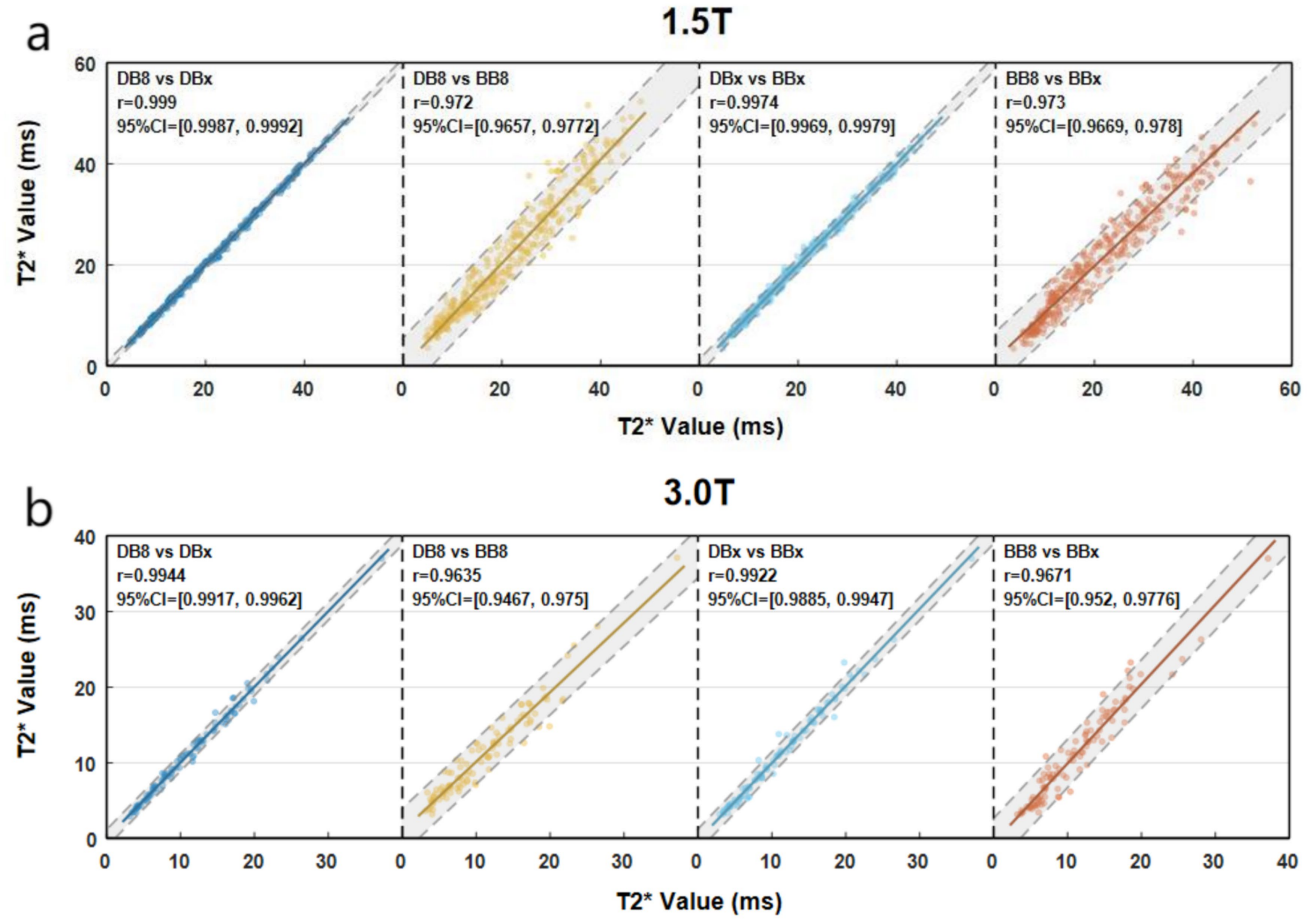
Scatter plots of myocardial T2* quantified by different methods (**a** vs. **b**, X axis corresponds to **a**, Y axis corresponds to **b**).

1.5 T group: 1.5 T-DB8-T2*, 1.5 T-DBX-T2*, BB8-T2* and BBx-T2* had no significant statistical difference (*p* = 0.575 > 0.05). DB8-T2* was highly correlated with DBx-T2* and BB8-T2* (*p* < 0.0001, r_s_ = 0.999, 0.972) (Figure 2). There was a strong correlation between DBx-T2* and BBx-T2* (*p* < 0.0001, r_s_ = 0.9974). There was a strong correlation between BB8-T2* and BBx-T2* (*p* < 0.0001, r_s_ = 0.973). 3 T group: DB8-T2*, DBx-T2*, BB8-T2* and BBx-T2* had no significant statistical difference (*p* = 0.764 > 0.05). DB8-T2* was highly correlated with DBx-T2* and BB8-T2* (*p* < 0.0001, r_s_ = 0.9944, 0.9635). There was a strong correlation between DBx-T2* and BBx-T2* (*p* < 0.0001, r_s_ = 0.9922). There was a strong correlation between BB8-T2* and BBx-T2* (*p* < 0.0001, r_s_ = 0.9671). The preliminary results showed that there was no significant difference between DB sequence and BB sequence in the quantification of myocardial iron load in TM patients, that is, DB sequence and BB sequence may replace each other in the quantification of myocardial iron load to a certain extent.

### 3.4 Bland–Altman consistency analysis of measurement results of different methods

**Figure 3:**
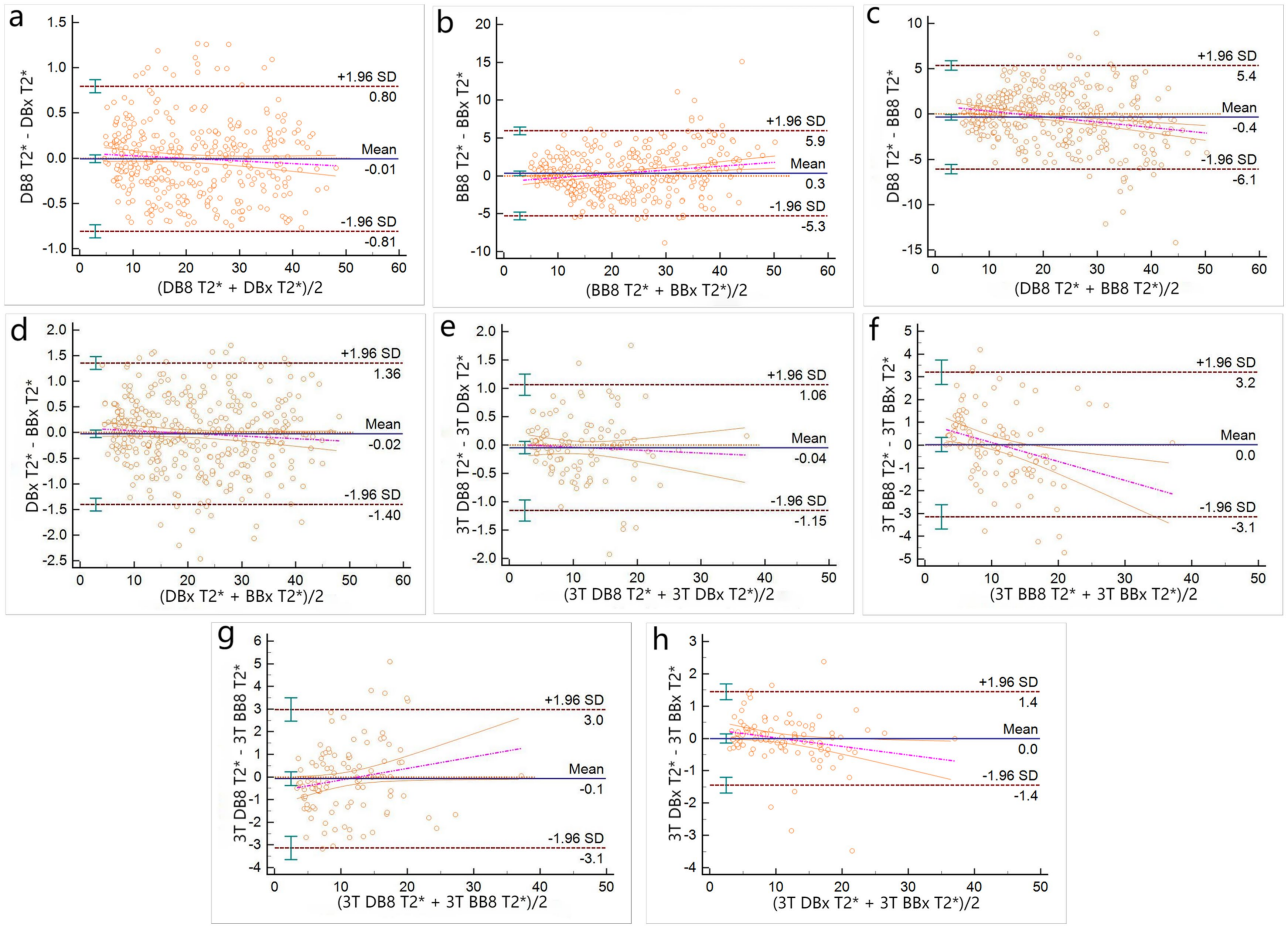
Bland–Altman plots of T2* measured by different methods (**a–d** for 1.5 T group, **e–h** for 3 T group).

1.5 T group: The Bland–Altman analysis between DB8-T2* and DBx-T2*, DBx-T2* and BBx-T2* were not statistically significant (*p* = 0.7926, 0.5281) (Figures 3a,b). There was no significant difference in proportion (the overall intercept and slope of the Bland–Altman regression equation were not significantly different from 0) (Table 2). Bland–Altman analysis between BB8-T2* and BBx-T2*, DB8-T2* and BB8-T2* were statistically significant (*p* = 0.0307, 0.021) (Figures 3c,d), and there were differences in proportion (Table 2). The results showed that DB8-T2* and DBx-T2*, DBx-T2* and BBx-T2* had good consistency, but it could not be considered that BB8-T2* and BBx-T2*, DB8-T2* and BB8-T2* had good consistency. At the same time, it also shows that DB technology is more stable than BB technology in quantifying myocardial T2*, that is, it is not susceptible to the influence of deviation signal.

**Table 2:** Bland–Altman agreement analysis of the results measured by different methods.

Comparison group	Cases (*N*)	95%CI number of cases (*N*) [%]	Regression equation	Equation population intercept 95%CI (P)	Equation population slope 95%CI (P)
1.5 T-DB8/DBx-T2*	359	13 (4.46%)	y = 0.05418–0.002844x	−0.03827 ~ 0.1466 (*p* = 0.2499)	−0.006748 ~ 0.001059 (*p* = 0.1527)
1.5 T-DBx/BBx-T2*	359	18 (5.01%)	y = 0.08393–0.0051x	−0.07487 ~ 0.2427 (*p* = 0.2993)	−0.01180 ~ 0.001596 (*p* = 0.1351)
1.5 T-BB8/BBx-T2*	359	9 (2.5%)	y = −0.7687 + 0.05165x	−1.3973 ~ −0.1400 (*p* = 0.0167)	0.02543 ~ 0.07787 (*p* = 0.0001)
1.5 T-DB8/BB8-T2*	359	13 (3.62%)	y = 0.9105–0.05974x	0.2719 ~ 1.5490 (*p* = 0.0053)	−0.08641 ~ −0.03307 (P < 0.0001)
3 T-DB8/DBx-T2*	106	6 (5.66%)	y = 0.01201–0.005140x	−0.2160 ~ 0.2400 (*p* = 0.917)	−0.02333 ~ 0.01305 (*p* = 0.5764)
3 T-DBx/BBx-T2*	106	6 (5.66%)	y = 0.2938–0.02663x	0.006545 ~ 0.5811 (*p* = 0.0451)	−0.04942 ~ −0.003831 (*p* = 0.0225)
3 T-BB8/BBx-T2*	106	6 (5.66%)	y = 0.9504–0.08342x	0.3177 ~ 1.5831 (*p* = 0.0036)	−0.1340 ~ −0.03288 (*p* = 0.0014)
3 T-DB8/BB8-T2*	106	5 (4.72%)	y = −0.6386 + 0.05127x	−1.2742 ~ −0.00308 (*p* = 0.0489)	0.0002305 ~ 0.1023 (*p* = 0.049)

3 T group: There was no significant difference in Bland–Altman analysis between DB8-T2* and DBx-T2*, DBx-T2* and BBx-T2*, BB8-T2* and BBx-T2*, DB8-T2* and BB8-T2* (*p* = 0.4197, 0.627, 0.9969, 0.851) (Figures 3e,f), but only between DB8-T2* and DBx-T2* there was no proportional difference (Table 2). The results indicate that DB8-T2* and DBx-T2* have good consistency, but it cannot be considered that DBx-T2* and BBx-T2*, BB8-T2* and BB8-T2* have good consistency. At the same time, it also shows that DB technology is more stable than BB technology in quantifying myocardial T2*, that is, it is not susceptible to the influence of deviation signal.

## 4 Discussion

In this study, myocardial T2* measured by DB CMR T2* and BB CRI T2* using different measurement methods was compared to investigate the difference of DB and BB T2* techniques at 1.5 T and 3 T in the assessment of myocardial iron load in patients with TM. The result indicated DB CMR T2* and BB CMR T2* can be interchangeable in the assessment of myocardial iron load in TM patients. Nevertheless, DB CMR T2* exhibits greater stability and reliability.

Single-breath-hold multi-echo BB CMR T2* technology can complete the acquisition of cardiac images in a single breath-hold, which has the characteristics of short scanning time and good reproducibility, and is widely used (19–27). However, the contrast between myocardium and blood pool in bright-blood technique is not high, which may affect the measurement accuracy. Subsequently, He et al. (27) developed the single-breath hold multi-echo DB CMR T2* technique by using the double-inversion prepulse technique, which could better define the boundary between the myocardium and the blood pool and improve the measurement accuracy.

The results showed that although there was no significant difference between DB CMR T2* and BB CMR T2*, there was a high positive linear correlation. This indicates that the two techniques may substitute for each other in the quantification of myocardial T2* to some extent, which is consistent with Khater and Ou et al. (20, 21), who stated that, the use of a blood suppression prepulse black blood technique had little effect on the calculated cardiac R2*/T2*.

However, when we performed the deep consistency analysis, it was found that the consistency of different methods was not the same. As can be seen from the results section, only DB8-T2* and DBx-T2* maintained good consistency in both 1.5 T and 3 T groups (Bland–Altman analysis showed no statistical difference, and there was no proportional difference), which proved that DB technique was more stable and reliable than BB technique in assessing myocardial iron load. This is similar to the findings of Liguori et al. (22). The 1.5 T similar results were reported by Smith et al. (23), who found a significant coefficient improvement (three folds) in intra and inter-observer variability with better inter-study reproducibility for BB T2* imaging compared to the conventional white blood sequence. In addition, our results partly indicate to some extent the imaging at 1.5 T is preferable as 3 T is less precise in patients with severe iron overload, which is similar with Meloni and Storey et al. (24, 25) who suggest that the iron-dependent component of R2* scales linearly with field strength over a wide range of tissue iron concentrations. The incidence of susceptibility artifacts may, however, also increase with field strength. The fact that 3-T MRI is not so reliable in very heavily loaded patients is important but provided it shows severe iron loading, it would indicate that the patients need to have intensive chelation and subsequently the testing would be more reliable once the patients have reduced that loading.

DB technology has better stability and reliability in quantitative assessment of myocardial iron load, and the reasons are as follows: (1) With BB technique, the blood pool is hyperintense, and the boundary between the myocardium with normal or mild iron deposition and the blood pool is blurred. It is difficult to define the myocardial boundary when delineating the region of interest, which may lead to inaccurate T2* measurement. Moreover, the blood pool shadow with high signal caused by cardiac motion and blood flow can be superimposed on the septal myocardium, forming blood pool artifact. At this time, the measurement error of T2* may also be aggravated by blood pool artifact (14, 15). (2) DB technique suppresses the blood pool signal by using the double inversion recovery prepulse technique to make the blood pool appear as no/low signal: a presaturation pulse is applied before the blood flow enters the imaging volume to presaturate the blood flow. When the RF pulse is applied when it flows into the imaging volume, since the longitudinal magnetization vector of the pre-saturated blood flow is very small, it almost does not produce MR Signal, so the blood flow shows low signal, while the surrounding tissue shows high signal, resulting in contrast (22). (3) Single breath-hold multi-echo GRE-T2* sequence cardiac imaging with DB technology, because the blood flow in the ventricular cavity is low signal, while the myocardial tissue without or with mild iron deposition is high signal, resulting in contrast to set off the ventricular septum, which is easy to manually draw the endocardial and epicardial boundaries, and facilitate accurate measurement of myocardial T2* (14, 15, 22). (4) In addition, due to the suppression of the blood pool signal by DB technology, there are fewer artifacts caused by the blood pool on the acquired images, which further improves the consistency of the measurement results, which may also be the main reason why DB technology is more stable in quantifying myocardial T2*.

The limitations of this study are as follows: (1) There is no actual myocardial iron concentration as the “gold standard” to evaluate the accuracy of different methods for quantifying myocardial iron load. However, due to its invasiveness, it is very challenging to collect myocardial iron concentration in every patient by cardiac biopsy technique. (2) Using DB and BB CMR, only the transverse axial papillary muscle level of the middle interventricular septum was used to assess the myocardial iron load in TM patients, and T2* values of each segment were not measured and compared according to the standard of the American Heart Association (AHA). However, in this study, based on the data of 359 patients from multiple centers, the comparison between the full signal and the cut-off signal of the same sequence, the comparison between different sequences, and the further investigation at 3 T field strength CMR are highly reliable. (3) When ROI was delineated by different methods, although the measurement results within and between observers were highly consistent, artificial measurement errors were inevitable. At present, the methods based on artificial intelligence (AI) image registration have gradually matured. Perhaps in the future, the ROI registration of AI can be relied upon to enhance the repeatability between the center and the observer and reduce errors. (4) In this manuscript, T2* measurements were performed at the mid-septal segment of the myocardium, which is a common and practical approach. However, full-segment analysis based on the AHA 17-segment model is increasingly recommended to capture spatial heterogeneity in myocardial iron distribution. (5) The study focuses solely on T2* imaging. However, native T1 mapping and extracellular volume fraction (ECV) are increasingly recognized as useful, complementary tools for tissue characterization, especially in differentiating iron overload from fibrosis. Theses may be the direction of future research.

## 5 Conclusion

DB CMR T2* technique and BB CMR T2* technique could replace each other in the quantification of myocardial iron load within a certain range of cardiac iron content. However, DB CMR T2* is more stable and reliable than BB CMR T2* in assessing myocardial iron load in TM patients. DB CMR T2* technique is recommended for the assessment of myocardial iron load in TM patients, especially when using 3 T MR Scanners. BB CMR T2* technique should not be used as the first choice for the assessment of myocardial iron load, especially when using 3 T MR Scanners.

## Data Availability

The original contributions presented in the study are included in the article/supplementary material, further inquiries can be directed to the corresponding authors.
